# A single lysergyl peptide synthetase assembles lysergic acid amides in *Aspergillus* species

**DOI:** 10.1371/journal.pone.0350650

**Published:** 2026-06-18

**Authors:** Samantha J. Fabian, Abigail M. Jones, Jessica L. Fuss, Daniel G. Panaccione

**Affiliations:** West Virginia University, School of Natural Resources and the Environment, Morgantown, West Virginia, United States of America; University of South Florida, USA

## Abstract

The agriculturally and pharmaceutically important lysergic acid amides ergonovine and lysergic acid α-hydroxyethylamide (LAH) are synthesized from a lysergyl-alanine precursor. In ergot-alkaloid producing fungi of the family Clavicipitaceae, lysergyl-alanine is assembled and then reduced to ergonovine by a complex of two monomodular nonribosomal peptide synthetases: lysergyl peptide synthetase 2 (Lps2) and Lps3. LAH is the major ergot alkaloid product of these fungi when the Lps2/Lps3 complex interacts with the Bayer-Villiger monooxygenase encoded by *easO*. An α/β hydrolase fold protein encoded by *easP* increases LAH accumulation but is not essential for LAH biosynthesis. Lps2 and Lps3 do not occur in the several species of *Aspergillus* (including *A. leporis*) that produce LAH and ergonovine. Instead, ergot alkaloid synthesis clusters of these *Aspergillus* species encode a novel two-module Lps gene, *lpsD*. We hypothesized the product of *lpsD* was functionally equivalent to the two separately encoded, monomodular enzymes of the Clavicipitaceae and tested this hypothesis by introducing *lpsD* of *A. leporis* into a strain of *Aspergillus fumigatus* that had been modified previously to accumulate lysergic acid as substrate. Introduction of *lpsD* resulted in accumulation of ergonovine as evidenced by high-performance liquid chromatography and liquid chromatography-mass spectrometry. The addition of the *A. leporis* allele of *easO* into the *lpsD*-transformed *A. fumigatus* strain led to accumulation of LAH. Introduction of a construct containing *easP* as well as *easO* into the *lpsD*-transformed *A. fumigatus* strain resulted in higher concentrations of LAH than in strains containing only *lpsD* and *easO*, consistent with previous studies in the Clavicipitaceae. The data support the hypothesis that ergot alkaloid-producing *Aspergillus* species independently evolved a single enzyme that serves the purpose of the two monomodular peptide synthetases of the Clavicipitaceae.

## Introduction

Ergot alkaloids derived from lysergic acid have a long, diverse, and continuing history as important agricultural and pharmaceutical chemicals. Ergotism (also call St. Anthony’s fire) resulting from ingestion of ergot alkaloid-contaminated rye and other grain crops infected by *Claviceps purpurea* caused significant human suffering and death throughout much of recorded history [1–3]. Accumulation of similar ergot alkaloids in common and important pasture grasses (including many species of ryegrass and fescue), as a result of symbiotic associations of the grasses with fungi in the genus *Epichloë*, affect agriculture in two ways: the ergot alkaloids deter and kill insect pests of the grass host but also significantly reduce the health and reproduction of grazing animals [2,4–7]. A third example of ergot alkaloid-producing fungi affecting agriculture can be observed in fungi in the genus *Metarhizium*. Several *Metarhizium* species produce ergot alkaloids of the lysergic acid amide class that contribute to their virulence against insects [8,9], a trait that affects their success as commercial biocontrol agents applied to agricultural crops [10,11]. *Claviceps* spp., *Epichloë* spp., and *Metarhizium* spp. are all members of the family Clavicipitaceae and share similar ergot alkaloid synthesis (*eas*) biosynthetic gene clusters [12,13]. In addition to their agricultural importance, lysergic acid-derived ergot alkaloids have impacted humankind significantly through their use as powerful pharmaceuticals for treating dementia, migraines, and Parkinson’s disease, among other conditions [12,14–16].

**Fig 1:**
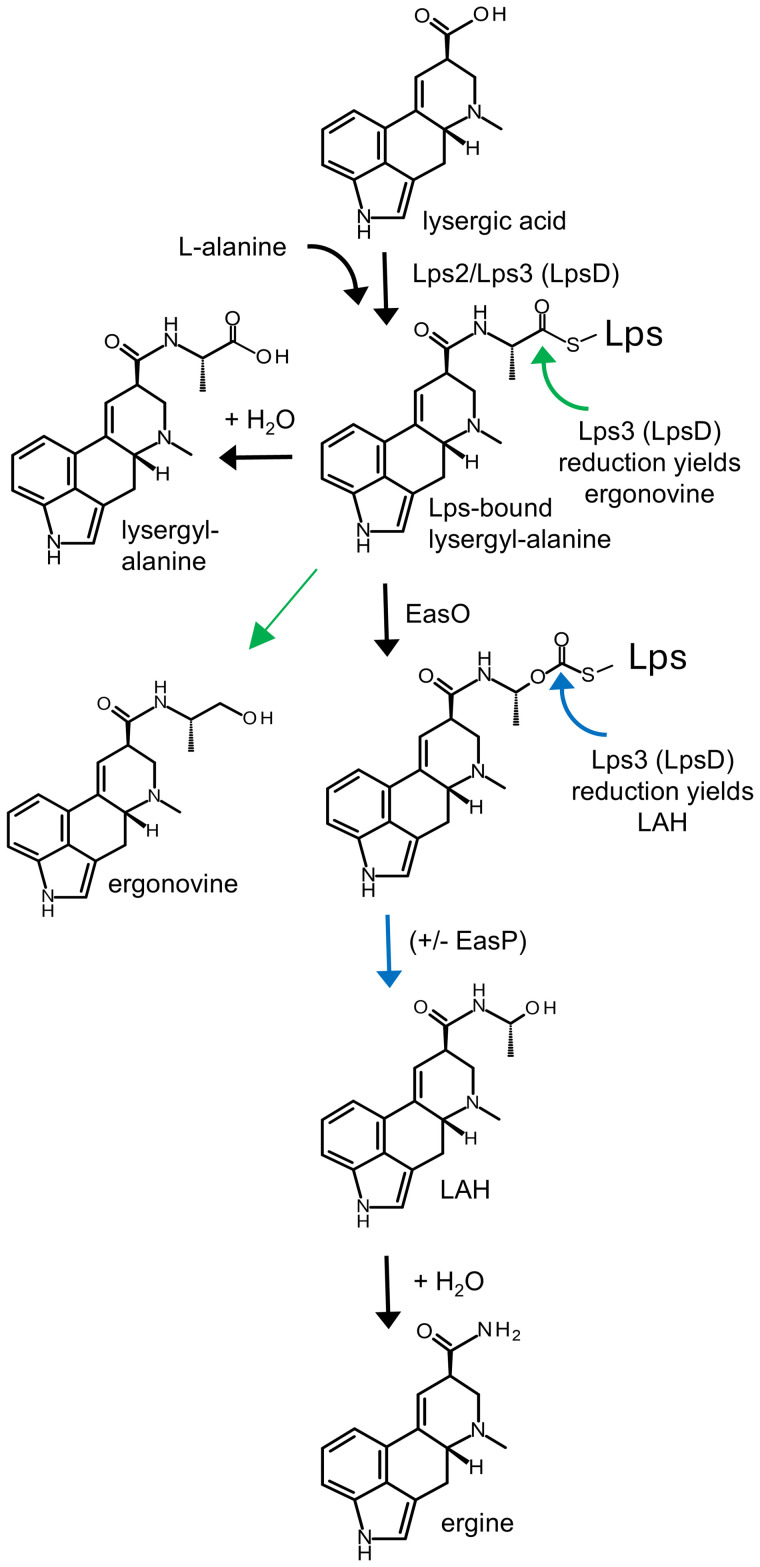
Biosynthetic pathway from lysergic acid to lysergic acid amides. Enzymes relevant to indicated steps are listed next to arrows. LpsD is listed parenthetically because it is the subject of the present study, as opposed to being established; EasP is listed parenthetically with +/- symbols because it is not essential but does increase yield. Green arrows represent reduction to ergonovine, whereas blue arrows show reduction to LAH. Abbreviations: Lps, lysergyl peptide synthetase; LAH, lysergic acid α-hydroxyethylamide.

Apart from fungi in the Clavicipitaceae, the ability to produce lysergic acid-derived ergot alkaloids was discovered recently in several species of *Aspergillus* [17]. *Aspergillus leporis*, *A. homomorphus*, and *A. hancockii* accumulate the lysergic acid amides ergonovine and lysergic acid α-hydroxyethylamide (LAH), along with its hydrolysis product ergine (Fig 1), with LAH being produced in the greatest abundance. *Aspergillus leporis* has been found associated with animal dung [18] and in the rhizospheres of metal-tolerant plants [19]. *Aspergillus homomorphus* has been isolated from soil [20] and stored millet [21], and *A. hancockii* was found in agricultural soils and on dried peas in Australia [22]. The *eas* clusters of these *Aspergillus* species contain genes similar to those of the Clavicipitaceous ergot alkaloid producers for pathway steps up through those required to produce lysergic acid; fungi in these two lineages differ, however, in the genes required to assemble lysergic acid into its amide derivatives [17].

**Fig 2:**
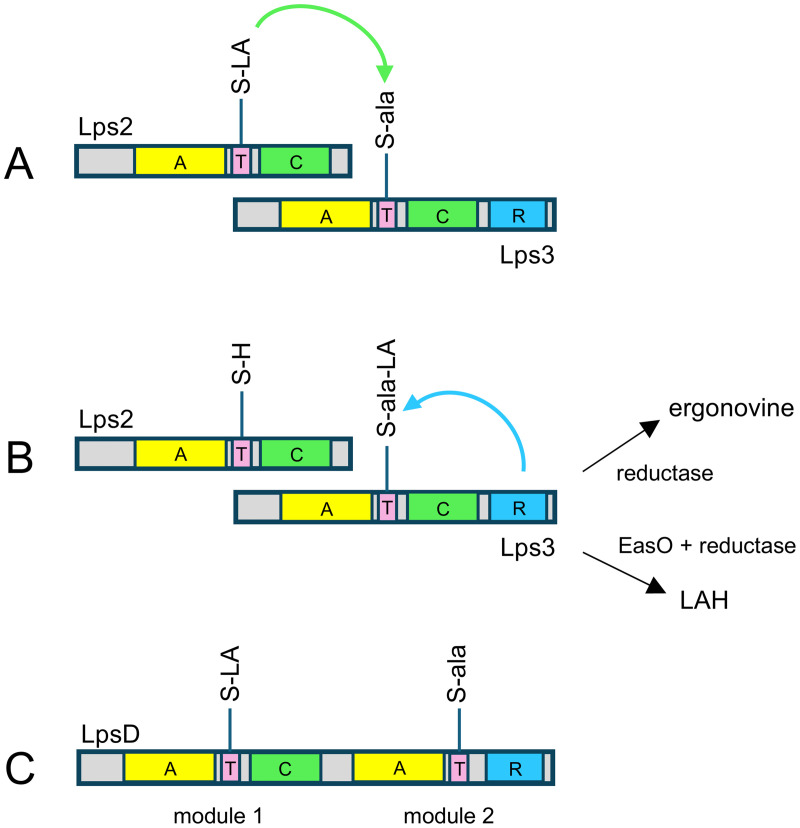
Structure and activity of lysergyl peptide synthetases, including the complex of Lps2 and Lps3 of ergot alkaloid producers of the Clavicipitaceae (panels A and B), and LpsD of the ergot alkaloid-producing *Aspergillus* species (panel C). Adenylation (A against a yellow background), thiolation (T against a purple background), condensation (C against a green background), and reductase (R against a blue background) domains are indicated in each multifunctional peptide synthetase. D-lysergic acid (LA) and L-alanine (ala) are depicted bound to 4’-phosphopanthetheine cofactors of thiolation domains. Green arrow in panel A depicts the condensation of the thioesterified lysergic acid residue from Lps2 to the amino terminus of the thioesterified L-alanine residue on Lps3. The blue arrow in panel B depicts the reduction of the carbonyl carbon of thioesterified lysergyl-alanine, to form ergonovine, or the product of EasO activity on Lps-bound lysergyl-alanine, to yield LAH.

Fungi in the Clavicipitaceae use an unusual combinatorial system comprised of two or three nonribosomal synthetases, depending on the organism, to assemble lysergic acid amides, ergopeptines, or a combination of lysergic acid amides and ergopeptines. Nonribosomal peptide synthetases have a modular structure with multiple domains in each module that catalyze consecutive steps in the assembly of a nonribosomally synthesized peptide [23,24]. A typical module contains an adenylation domain (to recognize an amino acid or carboxylic acid substrate and activate it by adenylation), a thiolation domain containing covalently bound 4’-phosphopantetheine (to which the activated substrate from the adenylation domain is transferred), and a condensation domain, which typically will form a peptide bound between the substrate bound to the thiolation domain and a neighboring substrate tethered to the thiolation domain of the next module. The carboxy terminus of a nonribosomal peptide synthetase often contains a domain responsible for release of the assembled peptide product. Different types of domains associated with release of peptide products include a thioesterase domain (to hydrolyze the peptide product from the final thiolation domain), a condensation domains (to cyclize the C-terminal substrate with the N-terminal or an internal substrate by peptide bond formation, thus releasing it from the enzyme), or a reductase domain to release the peptide product from the final thiolation domain via reduction. Lysergic acid amide producers in the Clavicipitaceae use a complex of two monomodular peptide synthetases, lysergyl peptide synthetase 2 (Lps2) which recognizes and acts upon lysergic acid, and Lps3, which recognizes and acts on L-alanine, to assemble lysergyl-alanine as an enzyme-bound intermediate (Fig 2). Lysergyl-alanine bound to the thiolation domain of Lps3 is then further modified to the lysergic acid amides ergonovine and/or LAH (Figs 1 and 2). Ergonovine is released from via reduction of the carbonyl carbon of the alanyl portion of lysergyl-alanine by the C-terminal reductase domain of Lps3 (Figs 1 and 2) [23]. Products of two additional genes contribute to synthesis of LAH from Lps-bound lysergyl-alanine: *easO* encodes a Bayer-Villiger monooxygenase (BVMO) that is essential for LAH biosynthesis [9,25], whereas *easP* encodes an α/β fold hydrolase protein that increases yield of LAH without being essential [26]. The BVMO EasO appears to insert an oxygen between the alpha and carbonyl carbons of the alanyl portion of Lps-bound lysergyl-alanine (Fig 1) [9]. In fungal mutants containing Lps2 and Lps3 but lacking EasO, ergonovine and lysergyl-alanine accumulated [9,25]. Steen et al. [9] hypothesized the diester substrate derived from EasO activity on lysergyl-alanine (Fig 1) is liberated from Lps3 through activity of the Lps3 reductase domain and that hypothesis was supported by recent gene editing of the active site of the reductase domain of Lps3 in *M. brunneum* [27]. In ergopeptine producers, the lysergic acid-activating enzyme Lps2 interacts with the trimodular peptide synthetase Lps1 (instead of Lps3) to initiate assembly of ergopeptines, derivatives of lysergic acid with three cyclized amino acids that vary between and define members of the ergopeptine family. Some members of the Clavicipitaceae contain genes encoding Lps1, Lps2, and Lps3 and produce both lysergic acid amides and ergopeptines through this combinatorial system [2,23].

The *eas* clusters of the three lysergic acid amide-producing *Aspergillus* species differ from those of the Clavicipitaceae in lacking individual, separate genes encoding Lps1, Lps2, or Lps3 and instead contain a single, novel Lps gene, *lpsD*, capable of encoding a two-module nonribosomal peptide synthetase (Fig 2C) [17]. In phylogenetic analyses conducted by Jones et al. [17], the combined adenylation and thiolation domains of the first module encoded in the *lpsD* gene formed a well-supported clade with those of Lps2 of the Clavicipitaceae. The second module of LpsD, however, was unlike those of Lps3 and clustered with adenylation/thiolation domains of other *Aspergillus* nonribosomal peptide synthetases in a clade well separated from the one containing Lps3 modules from members of the Clavicipitaceae, indicating no close evolutionary relationship with LpsC. LpsD does, however, contain a reductase domain at its carboxy terminus, and in this way appears to be functionally similar to Lps3 of the Clavicipitaceae (Fig 2C) [17]. Given this set of observations, we hypothesized that LpsD, the product of *lpsD*, was the functional equivalent of the two separately encoded monomodular peptide synthetases of the Clavicipitaceae (Lps2 and Lps3) and tested this hypothesis through introduction of *lpsD* from *A. leporis* into a previously engineered lysergic acid-accumulating strain of *A. fumigatus* [28]. Augmentation with LpsD was attempted with and without the addition of the LAH-associated genes *easO* and *easP* to test the role of LpsD in biosynthesis of LAH as well as ergonovine.

## Materials and methods

### Cloning of *lpsD* from *Aspergillus leporis*

The 7991-bp coding sequence of *lpsD* (containing a single intron) from *eas* cluster 1 of *A. leporis* strain NRRL 3216 [17], along with 433 bp of its 3’-untranslated region, was amplified as two separate but overlapping PCR products (because of its length) and joined to restore a single, functional gene (S2 Fig). The 5’-portion of the coding sequence also was joined to the 790-bp *easA/easG* promoter from *A. fumigatus* [28] by fusion PCR. The entire 9214-bp gene cassette was ultimately assembled, as described in detail below, by ligating the fragments into pUC18. Each of the PCRs described below consisted of a 20 µL volume comprised of 6 µL nuclease-free water, 10 µL Phusion green hot start II high-fidelity PCR master mix (Thermo Scientific, Waltham, MA), 2 µL of template DNA (approximately 10–50 ng), and 1 µL each of the respective forward and reverse primers diluted to 20 µM (Table 1). Whereas the annealing temperatures and extension times varied, all reactions followed the same general procedure: an initial denaturation at 98 °C for 30 seconds, followed by 35 cycles of denaturation at 98 °C for 15 seconds, primer annealing at the temperature indicated in Table 1 for 15 seconds, and DNA polymerase extension at 72 °C for a prescribed time interval (Table 1), followed by a final extension of 60 sec at 72 °C. PCR products were purified through the DNA Clean and Concentrator kit (Zymo Research, Irvine, CA). PCR with primer set 1 (Table 1) resulted in a fragment containing the 5’ region of *lpsD*, including the initiation codon and extending just beyond a unique *Sbf*I site would later become the junction of the two *lpsD* gene fragments. The fragment contained nucleotides 54,542–58,703 in GenBank accession SWBU01000165. The forward primer in this set contained 18 nt that overlapped with the *A. fumigatus easA/G* promoter to facilitate a subsequent fusion PCR (S2 Fig). Primer set 2 (Table 1) primed amplification of the *A. fumigatus easA/G* bidirectional promoter and contained the first 17 bp of *lpsD* at the 5’ end of the reverse primer, to facilitate the subsequent fusion PCR. The *lpsD* PCR product from primer pair 1 was joined to the *A. fumigatus easA/easG* promoter PCR product in a fusion PCR primed with primer set 3 (Table 1; S2 Fig). A unique *Nde*I restriction enzyme site was embedded in the 5’ end of the forward primer for the promoter fragment to facilitate cloning of the product as an *Nde*I*/Sbf*I-digested fragment into *Nde*I*/Sbf*I-digested pUC18. The unique *Sbf*I site found naturally in *lpsD* made a convenient site for eventual joining of the two separately amplified regions of the long gene. PCR with primer set 4 (Table 1) amplified a 5118-bp fragment containing the 3’ portion of the *lpsD* coding sequences along with 433 bp of 3’ noncoding region (including nucleotides 50,280–55,387 of GenBank accession SWBU01000165). The fragment contained the unique, internal *Sbf*I site near its 5’ end and a *Kpn*I site near its 3’ end. The *Kpn*I site had been built into the reverse primer which bound to the 3’ untranslated region of the gene. This second *lpsD* PCR product was digested with *Sbf*I and *Kpn*I and ligated with *Sbf*I*/Kpn*I*-*digested pUC18. The resulting *lpsD* fragments were ultimately joined in pUC18 by ligation at the *Sbf*I site to assemble a clone of the entire *lpsD* gene under the control of the *A. fumigatus easA/easG* promoter (S2 Fig).

**Table 1:** PCR primers, products, and conditions.

Primer pair	Primer sequences (5′ to 3′)	Product (length in base pairs)	Annealing temperature, extension time
1	GAGTAGGCACTCCGCACCATGTATGAGCAAGCTCC + GATGGCATAGCGAACGGTG	5’ portion of *lpsD* with extension^*a*^ to overlap with *A. fumigatus easA*/*easG* promoter (4180 bp)	65 °C, 120 s
2	CATTGCTTCTAATCCACCAAGTAC + GGAGCTTGCTCATACATGGTGCGGAGTGCCTACTC	*A. fumigatus easA*/*easG* promoter with extension to overlap with start of *lpsD* (807 bp)	63 °C, 60 s
3	^*b*^GCATCATATGCATTGCTTCTAATCCACCAAGTAC + CGACGGATGGACGCTCC	*A. fumigatus easA*/*G* promoter fused with first portion of *lps*D (4123 bp)	65 °C, 120 s
4	CGACGGATGGACGCTCC + CACAGGTACCCACGAGAAACGTCAAGGATCTG	3’ portion of *lpsD* (5118 bp)	65 °C, 140 s
5	CTTGATGAAGGCCAGATCGCTG + GCATAGTAGGCAAGTACAGCATTGG	*easO* with native promoter (3604 bp)	66 °C, 120 s
6	CCATCACAGGAGAATGCCTTTCG + GCATAGTAGGCAAGTACAGCATTGG	*easP* + *easO* with native bidirectional promoter (5042 bp)	66 °C, 120 s
7	GAGTAGGCACTCCGCACCATGTATGAGCAAGCTCC + CACAGGTACCCACGAGAAACGTCAAGGATCTG	*lpsD* coding sequences (7991 bp) plus 28 bp of flanking sequences	68 °C, 150 s

^*a*^ extension refers to incorporation of an additional 17–18 nt at the 5′ end of a primer to facilitate a subsequent fusion PCR

^*b*^ underlines indicate unique restriction sites added to primers to facilitate cloning of PCR products: *Kpn*I, GGTACC; *Nde*I, CATATG

**Cloning of *easO* and Combined *easO* and *easP* Fragments.** A 3604-bp PCR product (corresponding to nucleotides 20,768–24,371 of GenBank accession SWBU01000104) containing an *A. leporis* allele *easO* under the control of its native promoter (operationally defined as the 1396-bp immediately preceding the start codon) was amplified with primer pair 5 (Table 1; S3 Fig) and cloned as a blunt fragment into *Sma*I-digested pTW7705 [29,30] (Fungal Genetics Stock Center, Kansas State University, Manhattan, KS). A 5042-bp PCR product (containing nucleotides 19,330–24,371 of GenBank accession SWBU01000104) comprised of both *easO* and *easP* of *A. leporis* along with their native promoters (operationally defined as the entire 1396 bp between the start codons of the two divergently oriented genes) was amplified with primer pair 6 (Table 1; S3 Fig) and inserted as a blunt fragment into *Sma*I-digested pTW7705.

### Strain construction

Three types of strains were constructed in an *A. fumigatus* lysergic acid-accumulating background strain that has been previously engineered and described [28]. Briefly, the background strain was a previously engineered derivative of *A. fumigatus* isolate FGSC 1141 in which the native copy of *easA* had been knocked out [31] and genes *easA* and *cloA* from an ergovaline-producing *Epichloë* species were introduced resulting in an ergot alkaloid pathway terminating at lysergic acid [28]. For simplicity, the strain is referred to hereafter as *A. fumigatus* strain LA (for lysergic acid). The first type of novel strain engineered in the present study was transformed with *lpsD* from *A. leporis*, a second type contained *lpsD* and *easO* from *A. leporis*, and a third was augmented with the combination of *lpsD*, *easO*, and *easP,* all originating from *A. leporis.* All genes were introduced in integrative plasmids. Since previous CRIPSR/Cas-9 based gene knockouts in *M. brunneum* demonstrated that *easP* was not required for LAH synthesis but rather led to quantitative increases in LAH [26], the combination of *easP* and *lpsD* in the absence of *easO* was not constructed.

Mycelia from overnight cultures of *A. fumigatus* strain LA in 50 mL of malt extract broth [8] were incubated in 15 mL of filter-sterilized 0.7 M NaCl solution containing 60 mg Driselase (Sigma-Aldrich, Saint Louis, MO) and 1 g VinoTaste Pro (Crush2Cellar; Newberg, OR) to generate protoplasts. Protoplasts were purified and transformed as described previously [31,32]. To create strains containing *lpsD* of *A. leporis*, the *lpsD* plasmid constructed as described above was co-transformed into *A. fumigatus* strain LA along with pBCphleo [33] (Fungal Genetics Stock Center, Kansas State University, Manhattan, KS), conferring phleomycin resistance. Phleomycin-resistant transformants were initially screened by PCR with primer pair 1 (Table 1), and those yielding a positive fragment were rescreened with primer pair 7 (Table 1).

Strains of *A. fumigatus* containing *lpsD* and *easO* were engineered by introduction of the *A. leporis easO* construct prepared in pTW7705 into an *A. fumigatus* strain already containing *lpsD* and selecting for resistance to pyrithiamine encoded in pTW7705. Transformants were plated by mixing transformed protoplasts with 15 mL of molten (55 °C) pyrithiamine transformation medium (205 g sucrose, 2.5 g ammonium nitrate, 1 g Mg_2_SO_4,_ 0.5 g KH_2_PO_4_, 0.5 g K_2_HPO_4,_ 0.5 g KCl, 0.0 6g chloramphenicol and 7 g agarose per 1 liter of distilled water). A 15-mL layer of pyrithiamine transformation medium with 100 μg/mL of pyrithiamine was added as a second layer after the first layer of medium had solidified. Plates were incubated at 37 °C for 3 days. The strain of *A. fumigatus* containing *lpsD*, *easO*, and *easP* of *A. leporis* was engineered similarly by introducing the *A. leporis easO* + *easP*-containing pTW7705 construct into a strain of *A. fumigatus* previously transformed with *lpsD*. Transformants were checked for the presence of the introduced constructs by PCR with primer combinations 5 (for *easO*) and 6 (for the combination of *easO* and *easP*) (Table 1) and purified to nuclear homogeneity by culturing from single conidia.

### Analyses of ergot alkaloids

For routine analyses, ergot alkaloids were extracted from 14-day-old cultures of *A. fumigatus* strains on malt extract agar. An approximately 400-µL sample of fungal mycelium with associated conidia and agar medium was obtained by coring the fungal culture with the broader end of a 1000-µL pipet tip. The resulting cylinder of agar and fungal material was extracted in 400 µL of methanol. Twenty µL of sample was analyzed by HPLC with fluorescence detection. The HPLC apparatus consisted of a Waters model 600 pump with an in-line degasser, a model 717plus autosampler, and a Rainin (Woburn, MA) Fl2 fluorescence detector set at excitation and emission wavelengths of 310 nm and 410 nm, respectively. The solid phase was a Phenomenex (Torrance, CA) Prodigy ODS3 column of 5-µm particle size that was 150 mm in length and had a 4.6-mm inside diameter. The mobile phase was a multilinear, binary gradient from 5% acetonitrile + 95% 50 mM aqueous ammonium acetate to 75% acetonitrile + 25% 50 mM aqueous ammonium acetate over 55 min [8,9,34]. Ergot alkaloids were quantified relative to an external standard curve prepared from ergonovine (Sigma-Aldrich, St. Louis, MO) which contains the same fluorophore as the other investigated lysergic acid amides. For this reason, values for LAH and ergine must be considered as relative to ergonovine as opposed to absolute. Quantities of ergot alkaloids were expressed over a denominator of mass of fungus extracted that was estimated by counting conidia, which are the primary source of ergot alkaloids in *A. fumigatus* [35,36], and multiplying the number of conidia in the extract by the average mass of a conidium, 2.9 pg [35]. To compare quantities of ergot alkaloids statistically, variances were checked by a Brown-Forsythe test prior to running ANOVA and Tukey’s tests. A sample size of n = 6 was chosen for the quantitative study because comparisons of six replicates allowed for clear separation of means in a similar HPLC-based, quantitative study of lysergic acid amides in *M. brunneum* [26]. Statistical analyses were conducted with JMP version 18 (SAS; Cary, NC).

Ergot alkaloids also were analyzed through analysis on a Q-Exactive high-resolution liquid chromatography-mass spectrometry (LC-MS) system (Thermo Scientific, Waltham, MA). Ergot alkaloids were obtained in large quantities by washing conidia from the surface of 1-month-old malt extract agar cultures with 2 mL of methanol. We used a linear gradient of 5% acetonitrile plus 0.1% formic acid to 75% acetonitrile plus 0.1% formic acid over 10 min at a flow rate of 300 µL/min for the mobile phase. The column used for most analyses was a 150-mm length by 4.6-mm inside diameter, 2.6-µm particle size Kinetex Evo C18 column (Phenomenex, Torrance, CA); samples for analysis of lysergyl-alanine, however, were run on a 150-mm length by 2 mm inside diameter, 4-μm particle size Synergi Polar-RP C18 column (Phenomenex). Analytes were electrospray ionized in positive mode with a scan range of 100–400 m/z and fragmented with a normalized collision energy of 30%. Capillary temperature was set at 300 °C and spray voltage at 3.5 kV [17].

### Phylogenetic analyses

Rationale for inclusion of sequences for phylogenetic analysis of condensation domains (C domains) and reductase domains (R domains) was as follows. The C and R domains, as delimited for *A. leporis eas* cluster 1-encoded LpsD [27], were used as queries in blastp search of the NCBI database. Target organisms included representatives of all genera established as synthesizing ergonovine. For *Metarhizium*, which contains many sequenced ergonovine producers, *M. brunneum* ARSEF 3297 was selected as representative because its ergot alkaloids have been most intensively characterized. Similarly, *P. ipomoeae* Iasa13 was selected as the representative of the two sequenced *Periglandula* species. *Claviceps paspali* RRC1481 and *C. purpurea* 20.1 were chosen as representatives of the sequenced *Claviceps* species. The ergonovine producers among *Aspergillus* species contain only one sequenced representative per species. For analysis of the C domain, C domains from Lps3 were included (in addition to the C domain from Lps2 or LpsD) as well as C domains from modules 1 and 2 from the two ergopeptine producers (*C. purpurea* and *P. ipomoeae*) which are the only included taxa that contain Lps1. The carboxy-terminal C domains of *C. purpurea* and *P. ipomoeae* were excluded because they did not align well with the included C domains. For analysis of the R domain, additional related sequences were sought by conducting blastp or tblastn searches of each included taxon’s database with the R domain from Lps3 or LpsD of that same species as query. The top match encoded in each organism’s genome was included if it met the criteria of at least 30% identity over 70% query coverage [17]. Sequences were aligned with the default settings in Muscle as included in MEGA 11 [37] and trimmed by eye. Model tests in MEGA 11 indicated the Jones-Taylor-Thornton model with gamma distribution and invariant sites was the best approach for Maximum Likelihood analysis of the C domains and that the Le and Gascuel model with gamma distribution provided the best approach for the R domain dataset. Maximum Likelihood analyses were conducted with the indicated models in MEGA 11, applying 1000 bootstrap replications, and the tree with maximum likelihood is presented with bootstrap percentages at the nodes.

## Results and discussion

### LpsD catalyzes formation of ergonovine when introduced into a lysergic acid-producing host strain

**Fig 3:**
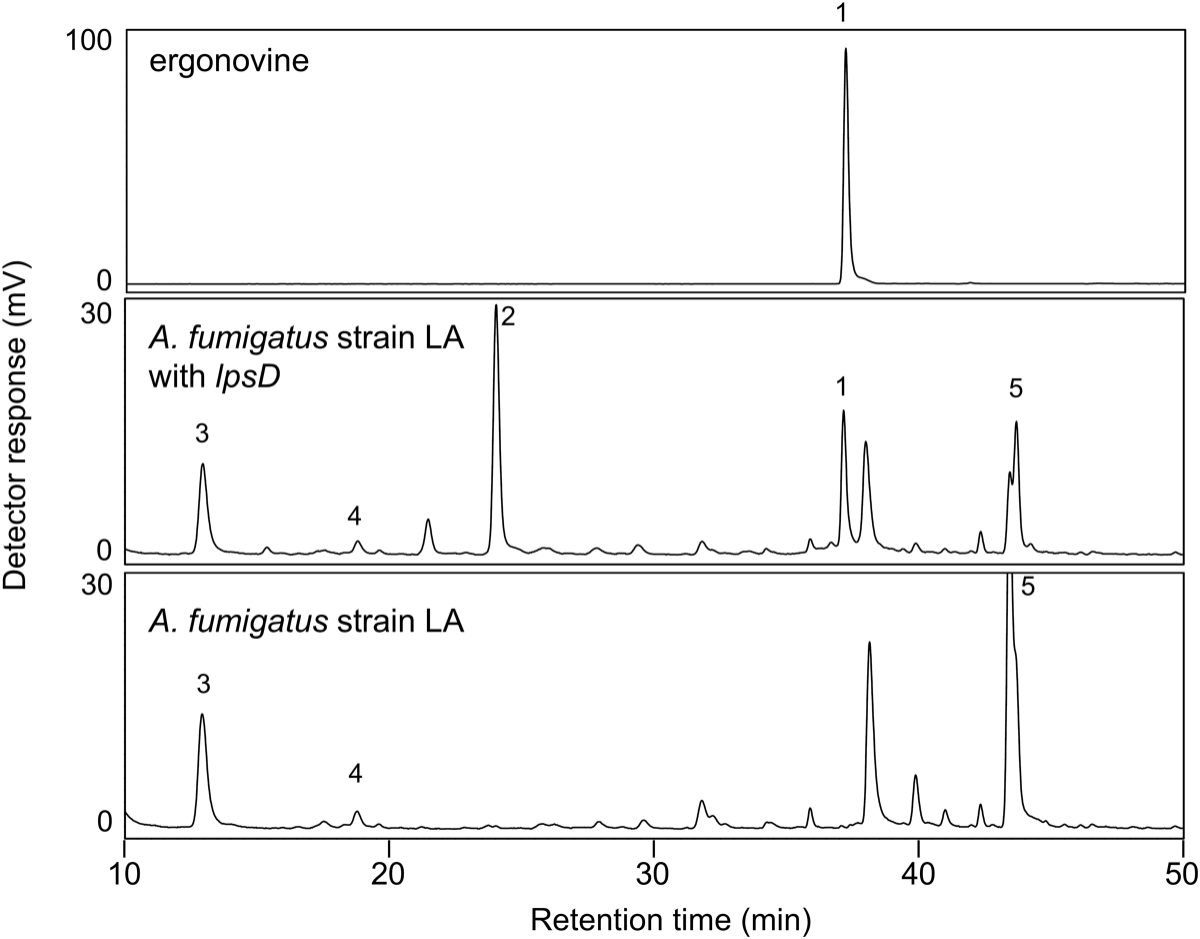
HPLC analyses of extract from *A. fumigatus* strain LA transformed with *lpsD* of *A. leporis* (middle panel) relative to ergonovine standard (above) or non-transformed host fungus *A. fumigatus* strain LA (below). Peak 1 represents ergonovine, peak 2 represents lysergyl-alanine, peak 3 corresponds to lysergic acid available as substrate in *A. fumigatus* strain LA, peak 4 is a stereoisomer of lysergic acid that forms in protic solvents (28), and peak 5 contains agroclavine which is the precursor to lysergic acid occurring in relatively high concentrations in *A. fumigatus* strain LA (28).

**Fig 4:**
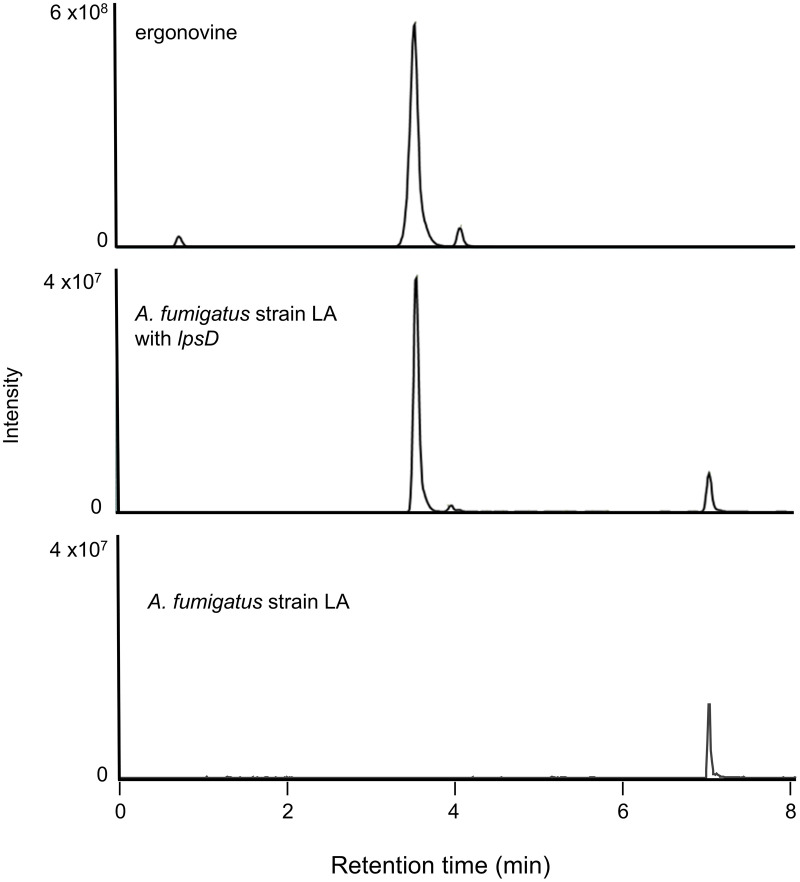
Extracted ion chromatograms of ions of m/z 326.1848–326.1878 (expected molecular ion for ergonovine is 326.1863 m/z) from a solution of ergonovine standard compared to methanol extracts of *A. fumigatus* strain LA transformed with *lpsD* of *A. leporis* and the non-transformed recipient, *A. fumigatus* strain LA.

Introduction of the *A. leporis lpsD*-containing construct into a strain of *A. fumigatus* previously engineered to accumulate lysergic acid resulted in 12 phleomycin-resistant colonies. Two transformants containing the construct, as evidenced by PCR analyses (S4 Fig), were selected for further study. The transformants were analyzed by HPLC with fluorescence detection, and the *lpsD*-transformed strains contained a novel analyte, as compared to peaks in the non-transformed recipient strain, eluting at the same time as authentic ergonovine standard (Fig 3). Other phleomycin-resistant transformants resulting from the attempted co-transformation of pBCphleo and *lpsD* (cloned into pUC18) lacked ergonovine, indicating that the accumulation of ergonovine in the selected transformants was not associated with introduction of pBCphleo alone (S5 Fig). The identity of the analyte in the *lpsD*-containing transformants as ergonovine also was supported by high-resolution LC-MS analyses. The *lpsD*-transformed strain contained an analyte yielding a molecular ion of 326.1863 (within 0.01 ppm relative to the calculated mass for protonated ergonovine) and eluting at the same time as ergonovine in this second chromatography system (Fig 4). The analyte from the *lpsD*-transformed *A. fumigatus* strain also fragmented in a manner consistent with the ergonovine standard (S6 Fig). A second novel analyte with fluorescence properties of a lysergic acid derivative and appearing in the HPLC chromatogram of the *lpsD*-transformed *A. fumigatus* strain eluted at 24 minutes (Fig 3), consistent with the elution time of lysergyl-alanine (the immediate precursor to ergonovine) observed in similar analyses previously [9]. In LC-MS analyses, the *lpsD*-transformed *A. fumigatus* strain contained an analyte with an m/z value of 340.1653 (within 0.8 ppm relative to the theoretical value for protonated lysergyl-alanine) that eluted at a time similar to lysergyl-alanine previously characterized in an *easO* knockout mutant of *M. brunneum* (S7 Fig) [9]. The fragmentation pattern of the m/z 340.1653 analyte was consistent with the fragmentation pattern of the analyte from the *easO* knockout of *M. brunneum* previously characterized as lysergyl-alanine (S8 Fig).

These data indicate that the single polypeptide product of *lpsD* catalyzes an analogous set of reactions as the two-enzyme system encoded by *lpsB* and *lpsC* that have been demonstrated previously to produce ergonovine from a lysergyl-alanine precursor in *C. purpurea* [23], a fungus species that has the combination of Lps2 and Lps3 but lacks the gene *easO* [38,39]. The metabolite profile of the *lpsD*-transformed strain of *A. fumigatus* also corresponds to that produced by the *easO* knockout of *M. brunneum* [9] in that the *lpsD* transformant accumulates the intermediate lysergyl-alanine along with ergonovine. One potential reason for the observation of detectable levels of lysergyl-alanine, the immediate precursor to ergonovine, in the *lpsD*-transformed *A. fumigatus* strain but not in the *C. purpurea* system is that the introduced LpsD may not have been functioning in a highly efficient manner in this artificial system and that lysergyl-alanine was being hydrolyzed off stalled Lps complex before the reductase domain could complete its reduction of ergonovine. Some fungi contain housekeeping thioesterases that remove stalled intermediates from peptide synthetases to free them up for subsequent rounds of catalysis [40]. An alternate hypothesis is that the observed lysergyl-alanine was derived from oxidation of the primary alcohol ergonovine back to a carboxylic acid by uncharacterized enzymes that may be inherent to *A. fumigatus*. Currently available data do not allow us to discriminate between these alternate hypotheses. A time course study may be informative, if oxidation of ergonovine is a significant source of lysergyl-alanine. Lysergyl-alanine would be expected to accumulate to a higher concentration in older cultures with a concomitant reduction in ergonovine concentration. The possibility that both hypothesized mechanisms operate simultaneously cannot be excluded, and continued production of lysergyl-alanine, along with ergonovine, from the peptide synthetase may confound interpretation of such a time course study.

### The combination of LpsD and EasO is sufficient for synthesis of LAH in a lysergic acid-accumulating background, and the addition of EasP increases LAH accumulation

The contribution of LpsD to LAH synthesis was studied in further transformation experiments that included the products of *easO* and *easP* from *A. leporis*. The gene *easO* from *A. leporis* and the combination of *easO* with *easP*, both from *A. leporis*, were introduced into the *A. fumigatus* strain that had previously been transformed with *lpsD* as part of an integrative plasmid conferring pyrithiamine resistance. Several pyrithiamine-resistant colonies were checked for presence of *easO* by PCR with primer pair 5 (Table 1), and two colonies containing the introduced *easO* gene fragment of 3.6 kb were selected for further analysis (S4 Fig). Additional pyrithiamine-resistant colonies were checked for presence of the *easO* and *easP-*containing*-*fragment by PCR, with one colony containing the expected PCR product of 5.0 kb (S4 Fig).

**Fig 5:**
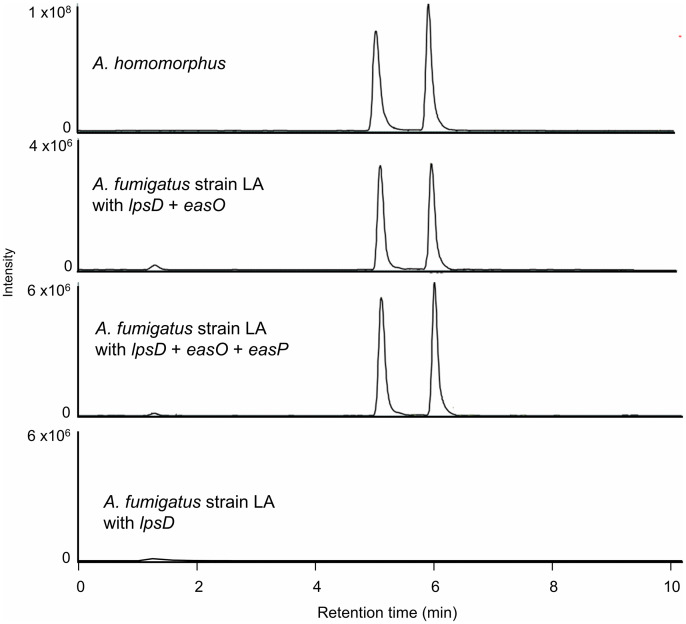
Extracted ion chromatograms of ions of m/z 312.1691–312.1721 (expected molecular ion for LAH 312.1706 m/z) from a reference extract of LAH-accumulating *Aspergillus homomorphus* [17] compared to extracts of *A. fumigatus* strain LA transformed with *lpsD* and *easO*, *A. fumigatus* strain LA transformed with the combination of *lpsD*, *easO*, and *easP* of *A. leporis*, or *A. fumigatus* strain LA without additional genes.

The *A. fumigatus* strain LA transformants containing *lpsD* and *easO* from *A. leporis* or the combination of *lpsD*, *easO*, and *easP* from *A. leporis* accumulated the lysergic acid amide LAH as demonstrated by LC-MS analyses. An isomeric pair of analytes of m/z 312.17 from strains containing *lpsD* + *easO* or *lpsD* + *easO* + *easP* eluted at the same time as the stereoisomeric pair of analytes corresponding to LAH in a reference extract from *A. homomorphous* (Fig 5) [17]. Certain lysergic acid derivatives stereoisomerize in protic solvents due to keto-enol tautomerization around the bond connecting the amide linkage to the four-membered ring system (Fig 1) [34,41]. The parent ion for a putative LAH peak of an *A. fumigatus* strain LA containing *lpsD* and *easO* had a m/z value of 312.1704, which corresponds to the calculated mass of protonated LAH with a mass accuracy of −0.8 ppm (S9 Fig). The transformant of *A. fumigatus* strain LA containing *lpsD*, *easO*, and *easP* contained a parent ion with m/z value of 312.1701, yielding a mass accuracy of −1.8 ppm relative to the expected value for protonated LAH (S9 Fig). Fragmentation patterns of the compounds produced in the modified *A. fumigatus* strains were consistent with that of an LAH reference extract obtained from *A. homomorphus* (S9 Fig) [17].

**Fig 6:**
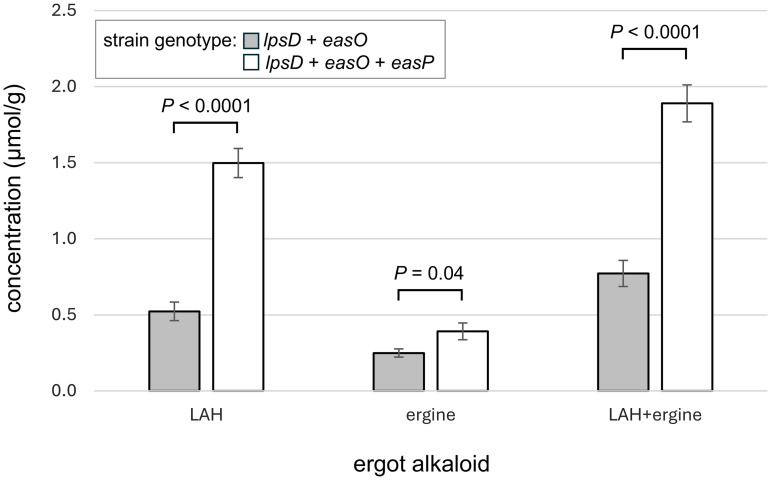
Mean quantities, relative to ergonovine, of LAH and ergine (or the sum thereof) accumulating in conidia extracted from cultures (n = 6) of *A. fumigatus* strain LA containing *lpsD* and *easO* or a strain containing the combination of *lpsD*, *easO*, and *easP* of *A. leporis*. Error bars represent standard error. Quantities are derived from peak areas relative to those of an external standard curve or ergonovine and thus must be considered ‘relative to ergonovine’ as opposed to absolute. *P* values associated with one-way ANOVAs are shown for individual species of ergot alkaloids or the combination of LAH and ergine.

Quantities of LAH were approximately 2.9-fold higher in a strain containing *easP* (along with *lpsD* and *easO*) relative to a strain containing *lpsD* and *easO* but lacking *easP* (*P* < 0.0001) (Fig 6). Ergine, the simple amide of lysergic acid, which forms spontaneously from LAH and other lysergic acid amides (Fig 1) [8,41,42], also was observed in the transformed strains of *A. fumigatus*. Considering the relationship of ergine to LAH, its concentrations in the transformed strains was considered alone and in combination with those of LAH. With the inclusion of ergine along with LAH, the combined lysergic acid amide accumulation was again significantly greater in the strain expressing *easP* in addition to *lpsD* and *easO* (*P* < 0.0001) (Fig 6). A similar increase in LAH accumulation of 2.4-fold was observed when the *lpsD* + *easO* + *easP*-containing strain was compared to a second *lpsD* + *easO*-containing transformant (S10 Fig). The increase in LAH accumulation resulting from the addition of *easP* is consistent with previous quantitative analyses of gene knockout mutants of the LAH-producing entomopathogen *M. brunneum* [26]. In that previous study, the wild-type strain accumulated LAH to an average concentration that was 2.8-fold higher than in strains in which *easP* was disrupted by a CRISPR/Cas9-based approach.

### Evolutionary and translational implications of LpsD from *Aspergillus* species compared to the multi-enzyme lysergyl peptide synthetase system of the Clavicipitaceae

**Fig 7:**
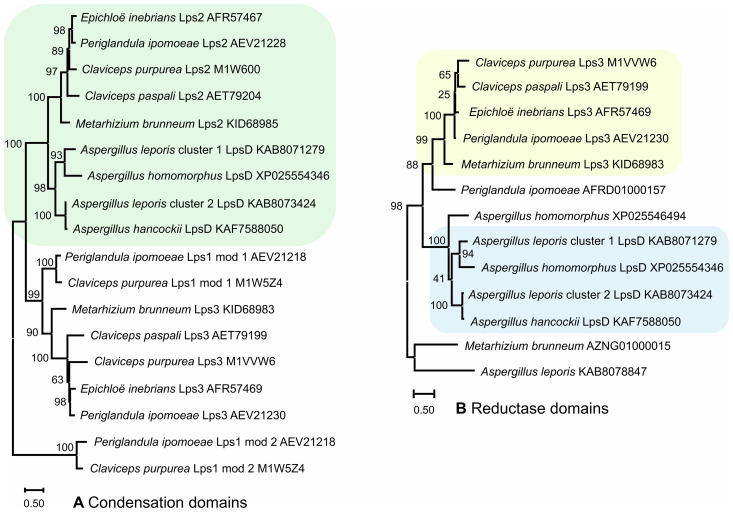
Maximum likelihood trees for (A) amino acid sequences corresponding to the C domains of LpsD from four *Aspergillus* species and Lps1 (modules 1 and 2), Lps2, and Lps3 from selected members of the Clavicipitaceae, and for (B) amino acid sequences corresponding to the R domains of LpsD from four *Aspergillus* species, from Lps3 from selected members of the Clavicipitaceae, and from homologs (identified by GenBank accession numbers) from the genomes of included fungi that contained at least 30% sequence identity over at least 70% query coverage with the R domain from the LpsD or Lps3 from that same organism. Trees presented have the greatest log likelihood of 1000 bootstrapped trees; bootstrap values are indicated at the corresponding nodes. The clade in (A) where the C domains of enzymes from the two lineages evolved from the same recent common ancestor are highlighted green, whereas clades containing R domains from *Aspergillus* species or the Clavicipitaceae which evolved from different recent ancestors are highlighted in blue and yellow, respectively. Scale bars represent changes/site.

The discovery of a single, alternate enzyme for lysergic acid amide synthesis in *Aspergillus* species as compared to the two-enzyme system documented previously for fungi of the Clavicipitaceae raises interesting questions about the evolution of these alternate approaches to synthesizing this important group of specialized metabolites. Previous phylogenetic analyses by Jones et al. [17] are relevant to this issue. The fact that the first adenylation and thiolation domains encoded by *lpsD* form a strongly supported clade with the lysergic acid-activating adenylation and thiolation domains encoded by *lpsB* of several lysergic acid amide-producing Clavicipitaceae indicates that *lpsD* and *lpsB* are derived from the same recent common ancestor. The second module of *lpsD*, however, was not closely related to the alanine-activating enzyme encoded by *lpsC* in similar analyses, indicating that *lpsC* is not derived from the same recent common ancestor as *lpsD* [17]. To further study the relatedness of LpsD with Lps2 and Lps3, we conducted additional phylogenetic analyses of the condensation (C) domains of all three enzymes and also of the reductase (R) domains, which are found only in LpsD and Lps3. The C domains of LpsD and Lps2 comprised a clade with 100% bootstrap support (Fig 7A), indicating that they were derived from the same recent common ancestor. The C domains of Lps3 formed a separate clade with the C domains from module 1 of Lps1 from ergopeptine producers. The R domains of LpsD and LpsC formed separate, well-supported clades with homologs from unspecified enzymes from fungi in their separate lineages (Fig 7B), indicating that the R domains from LpsD and LpsC do not share the same recent common ancestor. Overall, these phylogenetic analyses support previously published data from adenylation and thiolation domains that suggested module 1 from LpsD shares a recent common ancestor with Lps2 but module 2 of LpsD does not share a recent common ancestor with Lps3.

**Fig 8:**
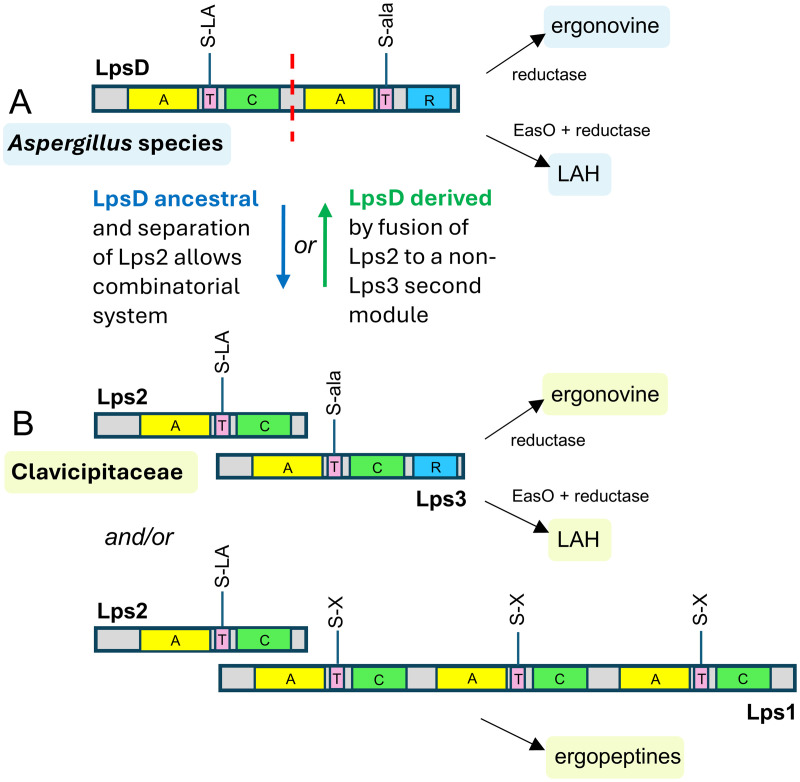
Graphical representation of the lysergyl peptide synthetases of *Aspergillus* species (A) and collective members of the Clavicipitaceae (B). Adenylation (A against a yellow background), thiolation (T against a purple background), condensation (C against a green background), and reductase (R against a blue background) domains are indicated in each module of each multifunctional peptide synthetase. D-lysergic acid (LA), L-alanine (ala), or a variety of amino acid substrates (X) are depicted bound to 4’-phosphopanthetheine cofactors on thiolation domains. The dashed red line in panel A indicates the point at which LpsD would have been truncated to yield Lps2 or the point where Lps2 would have been joined with a monomodular peptide synthetase to yield LpsD. The lack of establishment of the direction of evolution is indicated by the oppositely oriented blue or green arrows.

Based on the available data, we can only speculate on the order and process by which the alternate enzymatic approaches evolved (Fig 8). One possibility is that LpsD represents the ancestral state and that the fungi in the Clavicipitaceae (or an ancestor thereof) evolved the two-protein approach by separating the lysergic acid-activating module of LpsD (or an LpsD-like ancestor) from the remainder of the enzyme. Such an event may have been beneficial to those ergot alkaloid producers in the Clavicipitaceae because separating the first module (which would become Lps2) from the second module of LpsD allowed those fungi to use that first module in a combinatorial approach to make a more diverse array of lysergic acid derivatives. Combining Lps2 with the three-module peptide synthetase Lps1 would lead to synthesis of ergopeptines, while still allowing the combining of the separated Lps2 with the monomodular peptide synthetase Lps3 to restore the ability to make lysergic acid amides (Fig 8B). An alternative hypothesis would be that separate lysergyl peptide synthetases were the ancestral state. In this scenario the *Aspergillus* species that produce lysergic acid amides would have recombined a separate Lps2-like enzyme with a second monomodular peptide synthetase to create LpsD as a single polypeptide solution for making lysergic acid amides.

In support of LpsD being ancestral, the modular structure of Lps3 suggests that it may have been adapted to its role in lysergic acid amide synthesis from an ancestor that formerly served a different purpose in the Clavicipitaceae. Lps3 contains domains responsible for adenylating and thioesterifying L-alanine as well as a reductase domain to release lysergic amides from the Lps complex [9,23]. In between its adenylation/thiolation and reductase domains, Lps3 also has a C domain with no known purpose (Fig 8) [23]. Based on phylogenetics and conserved intron positions, Ortel and Keller [23] suggested that Lps3 and the first and third modules of Lps1 of *C. purpurea* evolved from a recent common ancestor. A comparison of the active sites of the C domain [24] in Lps3 in several ergot alkaloid producers compared to those in the first module of Lps1 reveals many more changes in amino acid sequences among different versions of Lps3 active sites relative to those in Lps1 (S11 Table). In a set of 14 Lps1 genes from sequenced ergopeptine producers occupying three genera, only two changes at one position were observed in the nine amino acid consensus sequence for the module 1 condensation domain active site. In contrast, in the Lps3 sequences from 16 ergonovine producers (spanning four genera) a total of nine types of changes (in 22 occurrences) were detected at four different positions relative to the nine amino acid consensus sequence for that condensation domain active site. Notably, in the Lps3 active sites, the conserved glycine residue of the canonical HHxxxDG motif [24] was always substituted by another amino acid: alanine, asparagine, threonine, or serine. The C domain in Lps3 may thus represent a remnant from an enzyme from which Lps3 evolved. There is only one C domain in LpsD (in a position corresponding to the C domain of Lps2) (Figs 2 and 8), and only one condensation reaction is required to assemble lysergyl-alanine, the immediate precursor to the lysergic acid amides.

Discovery of this single polypeptide lysergyl peptide synthetase also may have translational significance in simplifying ergot alkaloid pathway reconstruction. For example, Wong et al. [43], recently reported reconstitution of the ergot alkaloid through lysergic acid in yeast, and Xiao et al. [44] engineered a highly efficient lysergic acid-producing strain of *A. oryzae*. The addition of *lpsD* from *A. leporis* to either of these expression hosts would yield a strain capable of producing ergonovine. The addition of *lpsD* and *easO* would yield an LAH producer. A single lysergyl peptide synthetase should be easier to engineer into such heterologous expression systems than a two-enzyme lysergyl peptide synthetase system. We speculate that a single enzyme may also function more efficiently than a two-component system in which the two separately expressed enzymes would have to associate with one another properly in a heterologous host.

## Conclusions

Although ergot alkaloid producers in the genus *Aspergillus* and the family Clavicipitaceae are capable of producing the same lysergic acid amides, our data demonstrate that the biosynthetic processes differ between the fungi in the two lineages. Our results support the hypothesis that the ability to produce lysergic acid evolved once, but the ability to produce amide derivatives of lysergic acid evolved independently in *Aspergillus* species and the Clavicipitaceae. Further analyses of the lysergyl peptide synthetase genes of these fungi may provide information on the evolution of the two alternate approaches to lysergic acid amide synthesis. Moreover, *lpsD* may be a genetic resource with biotechnological value.

## Supporting information

S1 Fig Raw images. Original gels supporting S4 Fig.
(TIF)

S2 Fig Strategy for PCR-amplifying and cloning of *lpsD* from *Aspergillus leporis.* Descriptions of reactions are provided in the Material Methods section of the primary article and primer sequences and conditions are provided in Table 1 of the primary article. Pro, promoter between *easA* and *easG* of *Aspergillus fumigatus*.
(TIF)

S3 Fig Strategy for PCR-amplifying *easO* and the combination of *easO* and *easP* from *Aspergillus leporis* genomic DNA. Fragments were cloned as blunt end products into pTW7705. Descriptions of reactions are provided in the Material Methods section of the primary article and primer sequences and conditions are provided in Table 1 of the primary article.
(TIF)

S4 Fig PCR amplification from introduced fragments in transformants of *A. fumigatus* strain LA. Primer sequences and PCR conditions are provided in Table 1 of the primary article. Sizes of relevant fragments from *Bst*EII-digest bacteriophage λ are provided in kb; the 13.2-kb fragment comes from the 5.7-kb and 8.5-kb fragments annealing at their cos sites.
(TIF)

S5 Fig Lack of ergonovine in three phleomycin resistant transformants, indicating a lack of association of the selectable marker plasmid pBCphleo and ergonovine accumulation in *A. fumigatus* strain LA. Peaks for lysergic acid (and its stereoisomer) and agroclavine, inherent to the recipient strain *A. fumigatus* strain LA (Fig. 3) are labeled.
(TIF)

S6 Fig Fragmentation patterns of molecular ion resulting from commercially available ergonovine relative to that from an extract of *A. fumigatus* strain LA expressing *lpsD* of *A. leporis.* (TIF)

S7 Fig Extracted ion chromatograms of ions of m/z 340.1641–340.1671 (expected molecular ion for lysergyl-alanine 340.1656 m/z) related to accumulation of lysergyl-alanine. Labeled panels contain extracts from an *easO* knockout of *Metarhizium brunneum* previously established to contain lysergyl-alanine compared to extracts of *A. fumigatus* strain LA transformed with *lpsD* of *A. leporis* and the non-transformed recipient strain *A. fumigatus* LA.
(TIF)

S8 Fig Fragmentation pattern of molecular ion with the m/z of lysergyl-alanine from an extract of an *easO* knockout of *M. brunneum* compared to that of *A. fumigatus* strain LA transformed with *lpsD* of *A. leporis.* (TIF)

S9 Fig Fragmentation pattern of molecular ion with the m/z of LAH from an extract of *A. homomorphus* previously characterized as containing LAH [17] compared to extracts of *A. fumigatus* strain LA containing *lpsD* and *easO* or *A. fumigatus* strain LA transformed with the combination of *lpsD easO* and *easP* of *A. leporis.* (TIF)

S10 Fig Mean quantities, relative to ergonovine, of LAH and ergine (or the sum thereof) accumulating in conidia extracted from cultures (n = 6) of a second strain (relative to the strain shown in Figure 7 of the main manuscript) of *A. fumigatus* strain LA containing *lpsD* and *easO* compared to the strain of *A. fumigatus* strain LA containing the combination of *lpsD easO* and *easP* of *A. leporis.* Error bars represent standard error. Quantities are derived from peak areas relative to those of an external standard curve or ergonovine and thus must be considered ‘relative to ergonovine’ as opposed to absolute. *P* values associated with one-way ANOVAs are shown for individual species of ergot alkaloids or a combination thereof.
(TIF)

S1 Table Variation among active sites of condensation domains of lysergyl peptide synthetases. (TIF)

S1 File Raw data. **Data from which means and standard errors graphed in Fig 6 and** **S10 Fig** **are derived.** Data are supplied in a Microsoft Excel file with separate tabs for Fig 6 and S10 Fig.
(XLSX)
