# Supplementary figures and images for: A single lysergyl peptide synthetase assembles lysergic acid amides in Aspergillus species
Source: PLoS One. 2026 Jun 18;21(6):e0350650. doi: 10.1371/journal.pone.0350650 (PMC13278392; doi:10.1371/journal.pone.0350650)

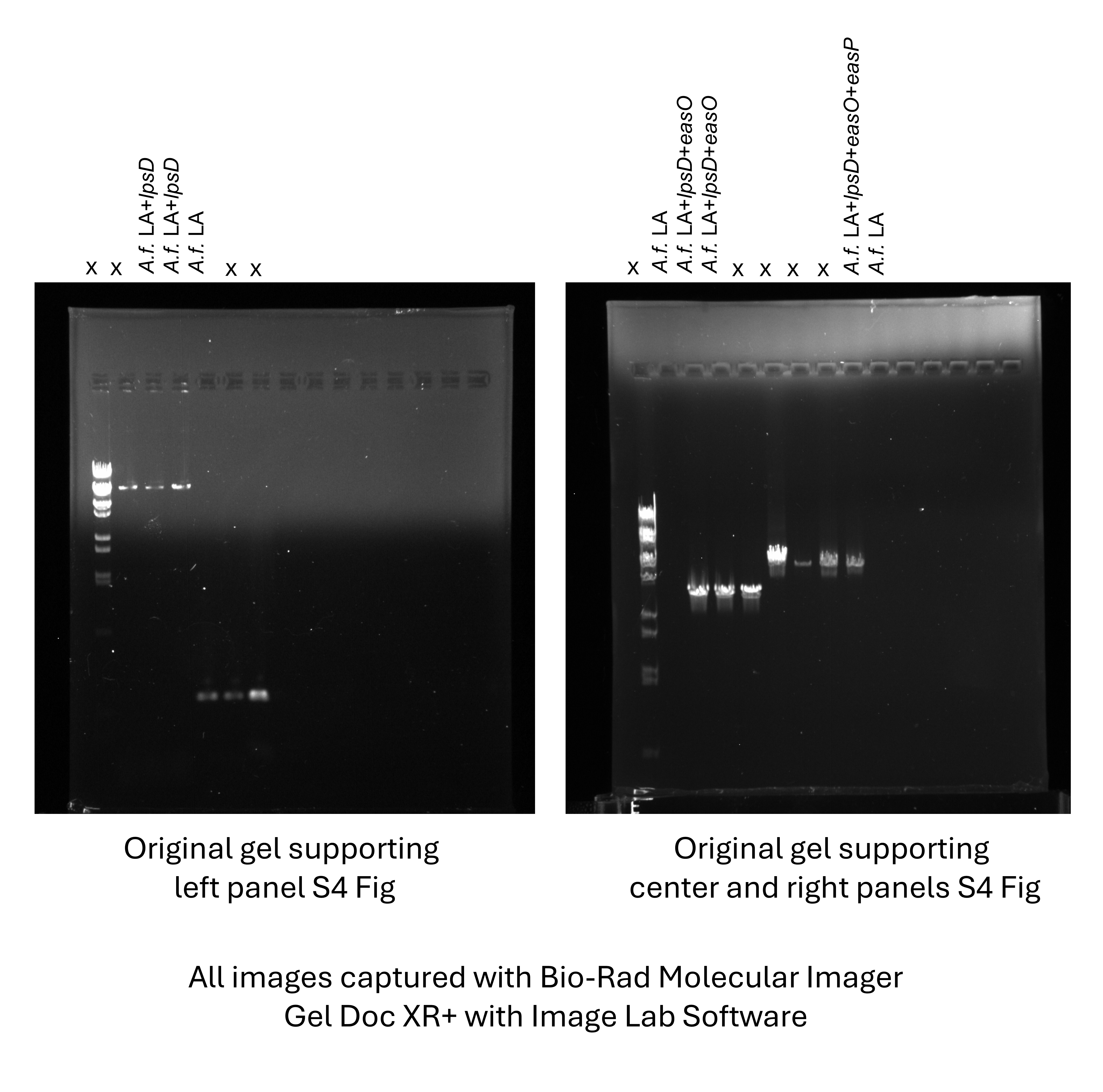

Supplement: S1 Fig — Original gels supporting S4 Fig. (TIF) [file pone.0350650.s001.tif]

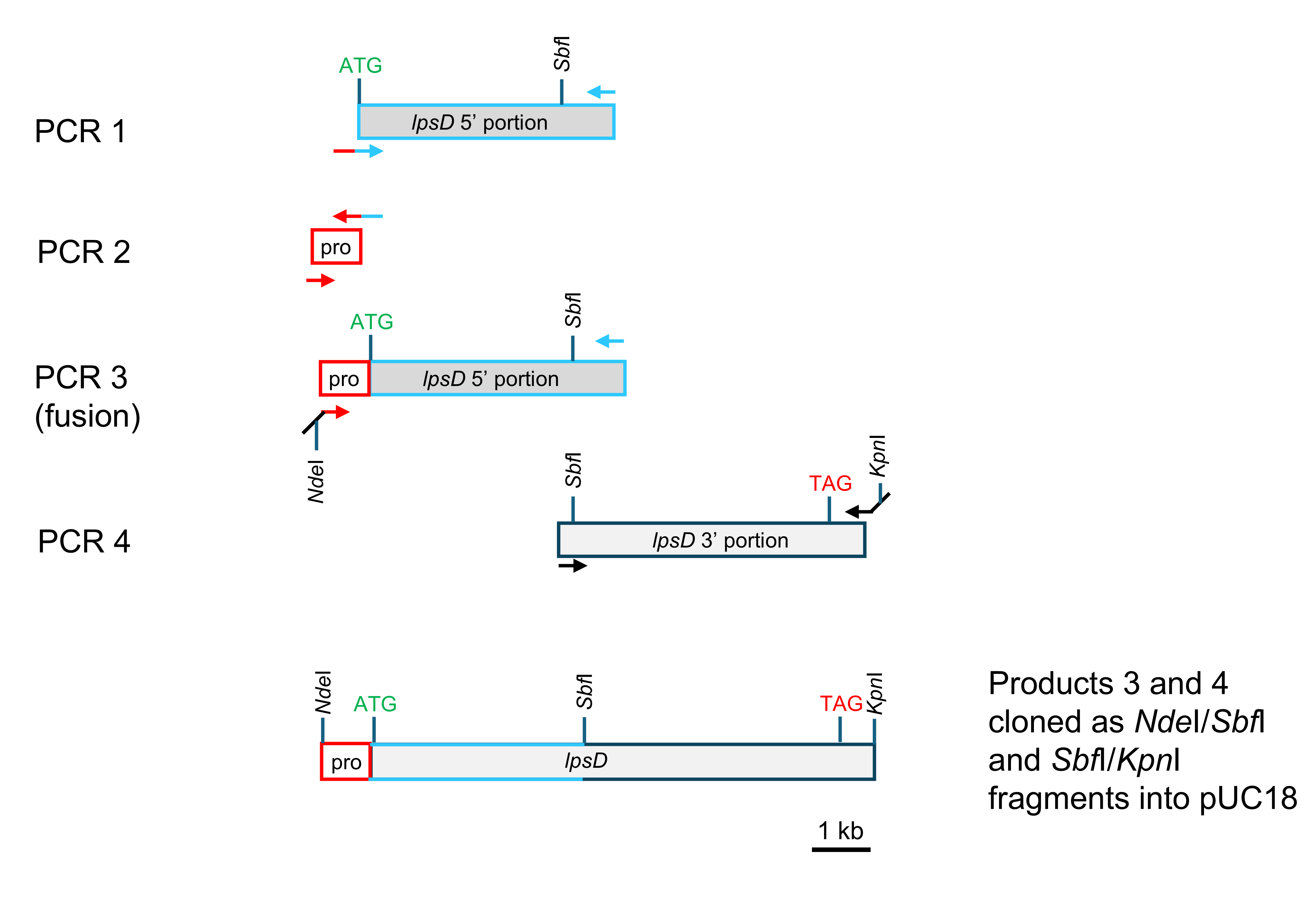

Supplement: S2 Fig — Descriptions of reactions are provided in the Material Methods section of the primary article and primer sequences and conditions are provided in Table 1 of the primary article. Pro, promoter between easA and easG of Aspergillus fumigatus. (TIF) [file pone.0350650.s002.tif]

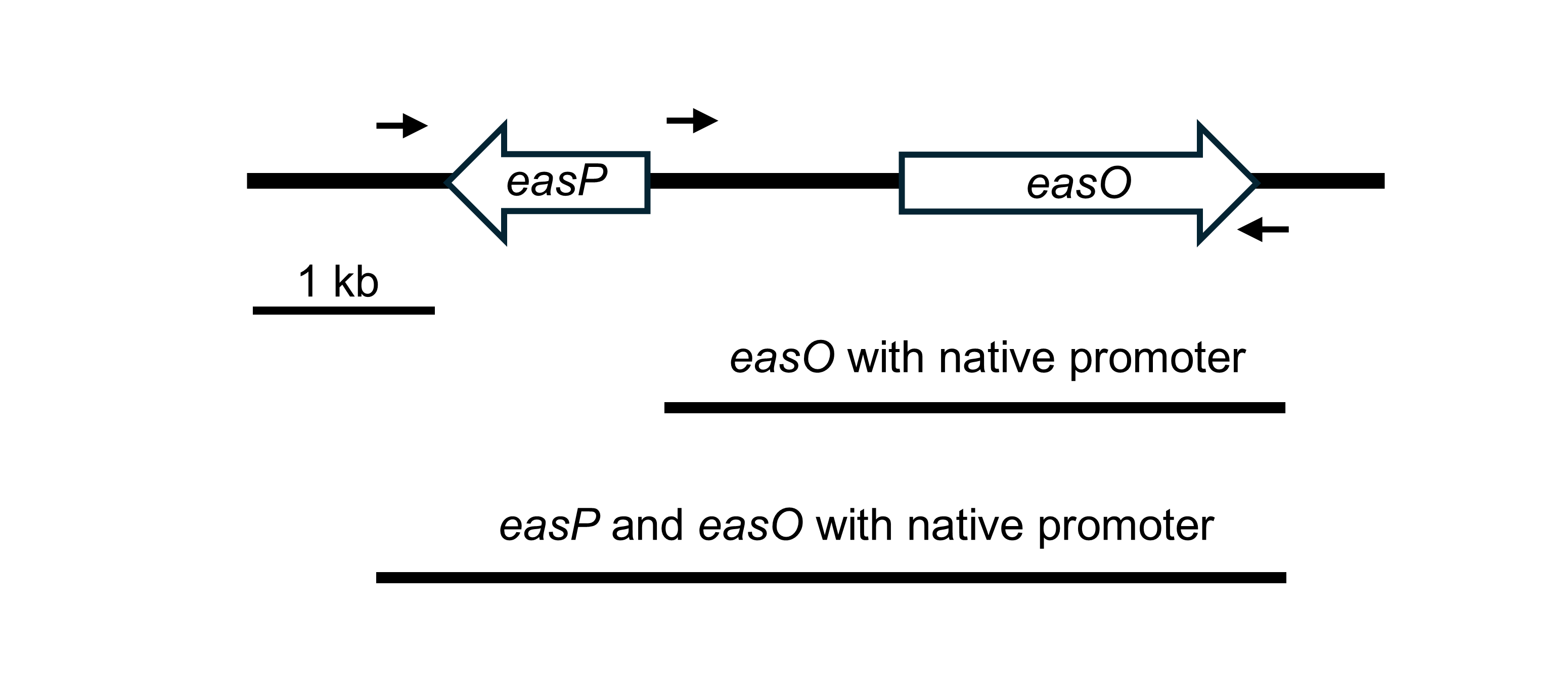

Supplement: S3 Fig — Fragments were cloned as blunt end products into pTW7705. Descriptions of reactions are provided in the Material Methods section of the primary article and primer sequences and conditions are provided in Table 1 of the primary article. (TIF) [file pone.0350650.s003.tif]

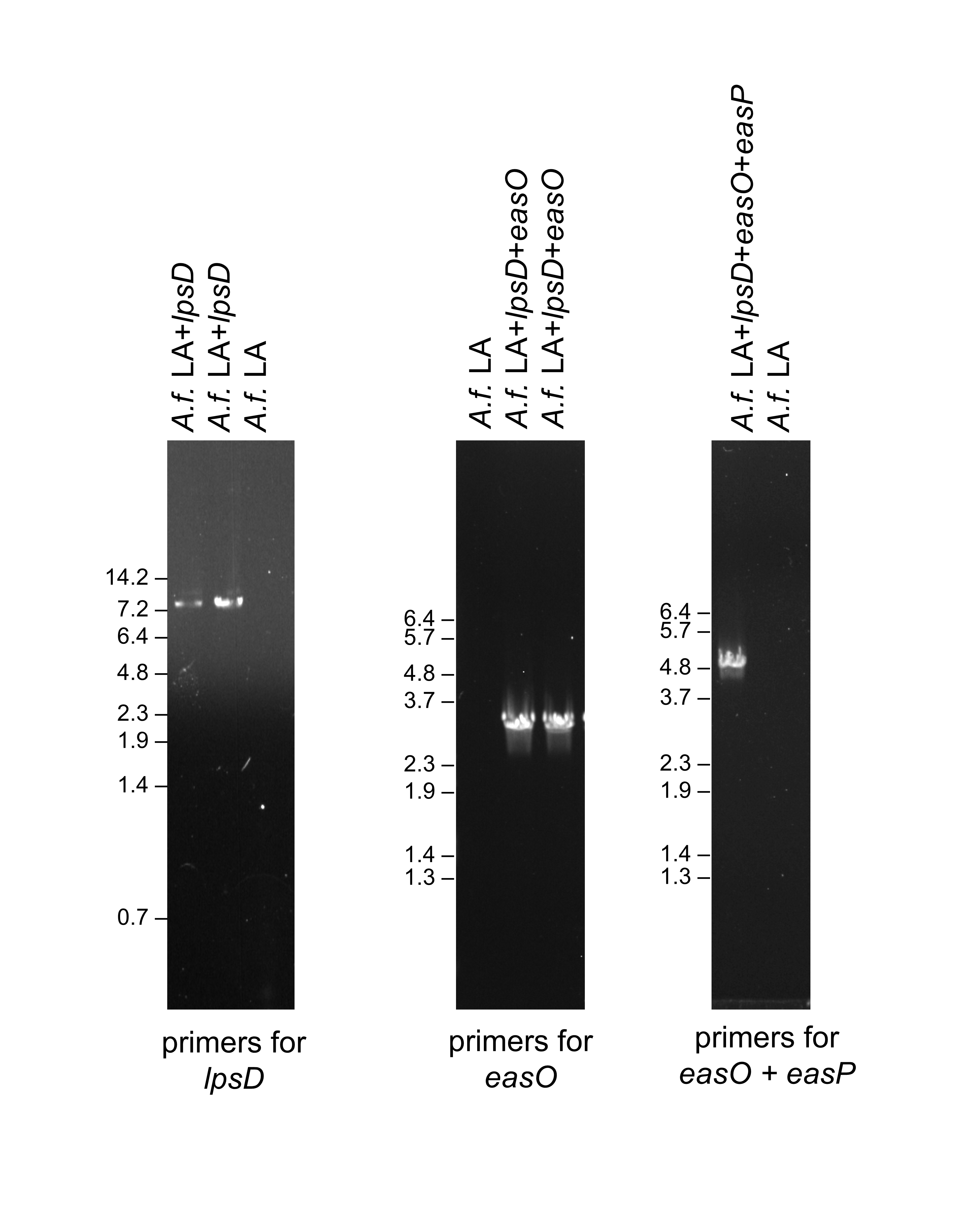

Supplement: S4 Fig — Primer sequences and PCR conditions are provided in Table 1 of the primary article. Sizes of relevant fragments from BstEII-digest bacteriophage λ are provided in kb; the 13.2-kb fragment comes from the 5.7-kb and 8.5-kb fragments annealing at their cos sites. (TIF) [file pone.0350650.s004.tif]

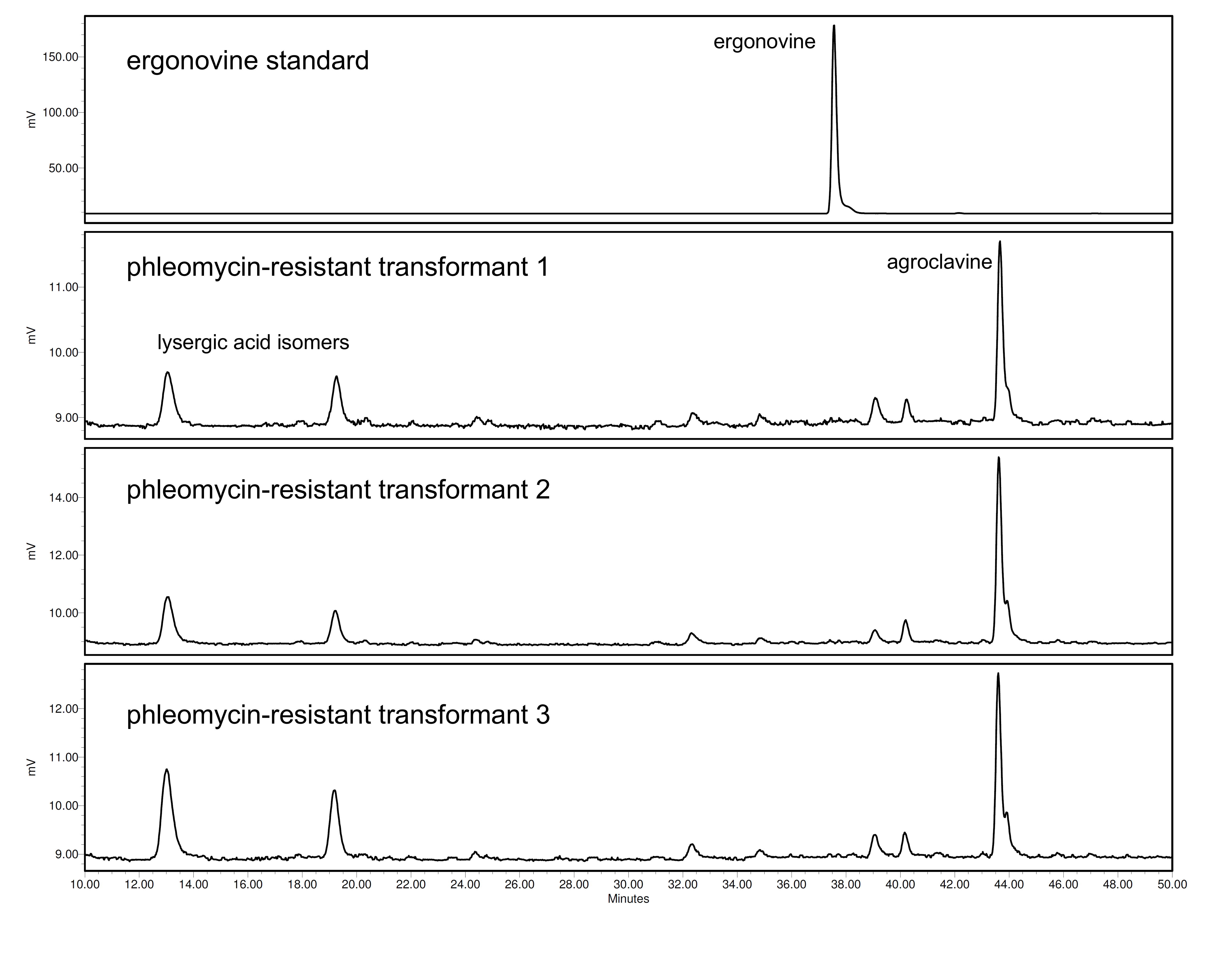

Supplement: S5 Fig — Peaks for lysergic acid (and its stereoisomer) and agroclavine, inherent to the recipient strain A. fumigatus strain LA (Fig. 3) are labeled. (TIF) [file pone.0350650.s005.tif]

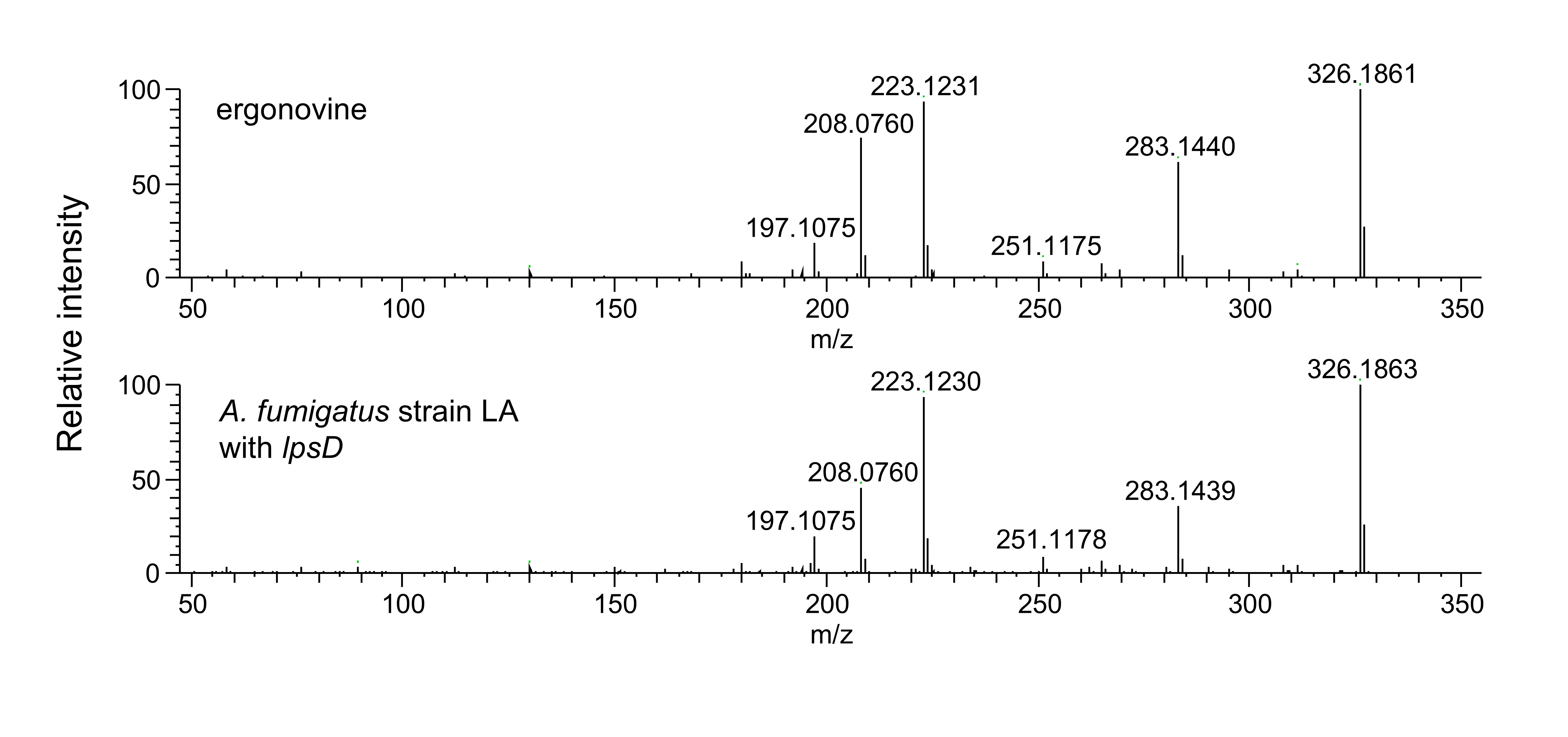

Supplement: S6 Fig — (TIF) [file pone.0350650.s006.tif]

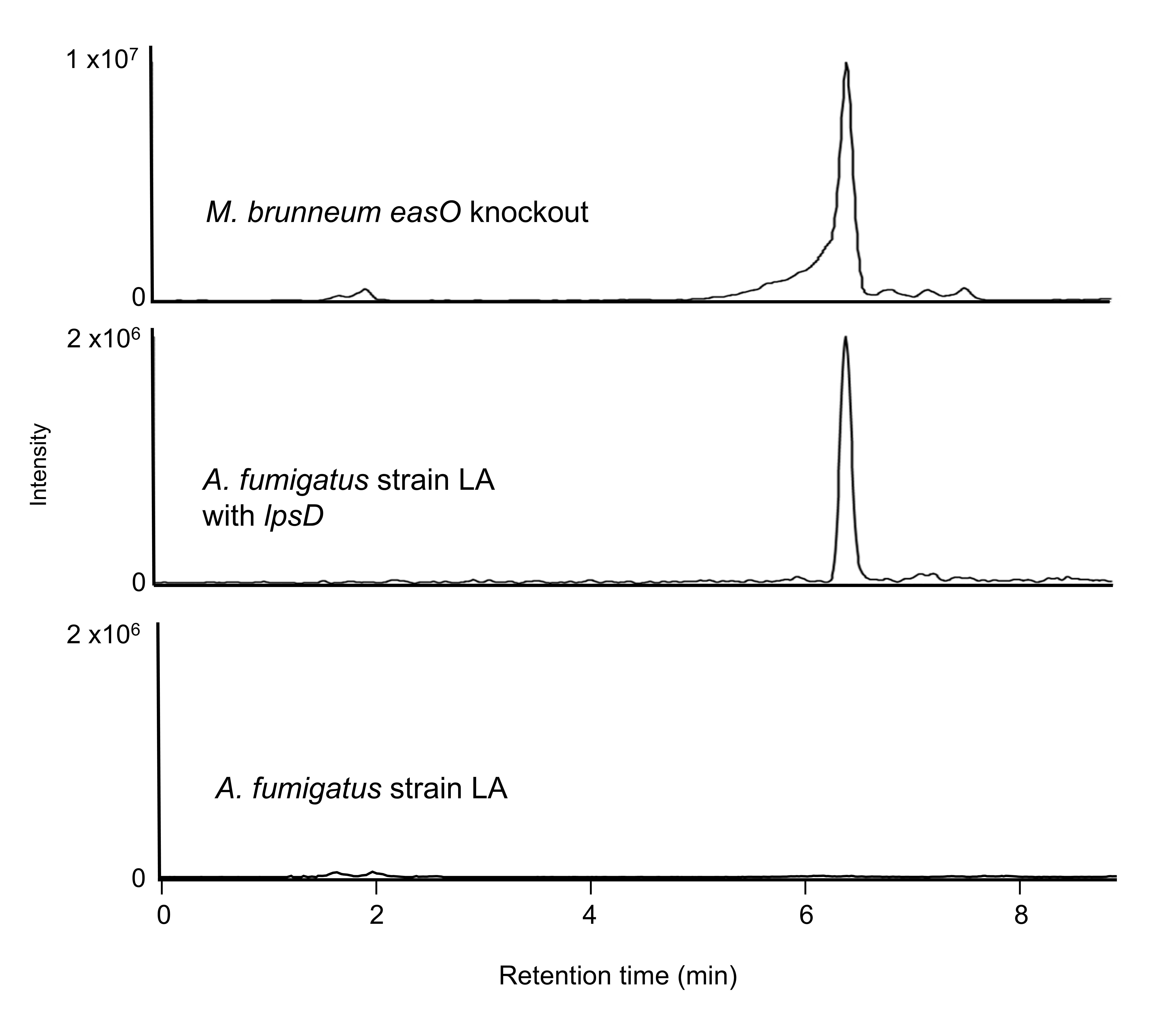

Supplement: S7 Fig — Labeled panels contain extracts from an easO knockout of Metarhizium brunneum previously established to contain lysergyl-alanine compared to extracts of A. fumigatus strain LA transformed with lpsD of A. leporis and the non-transformed recipient strain A. fumigatus LA. (TIF) [file pone.0350650.s007.tif]

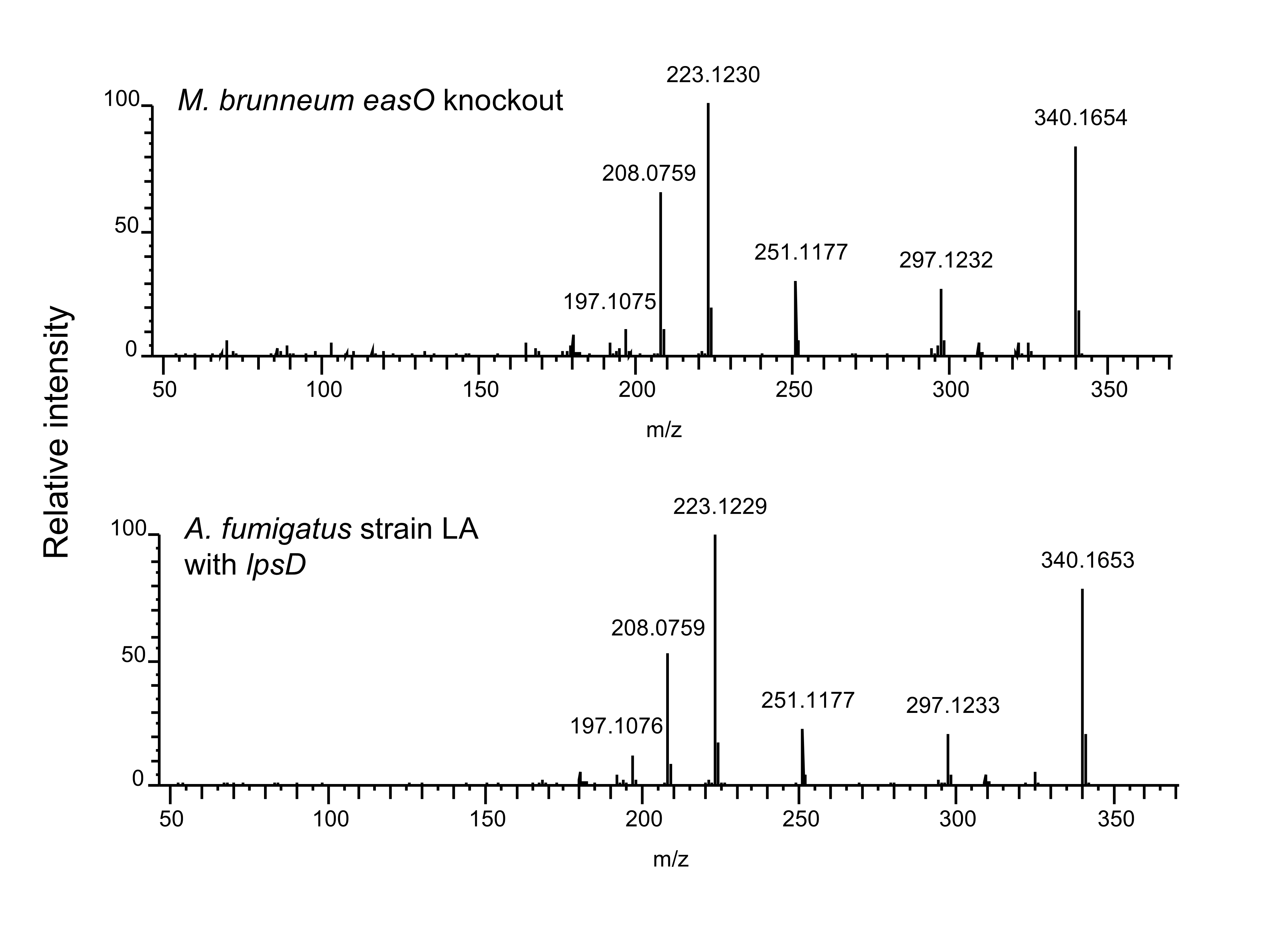

Supplement: S8 Fig — (TIF) [file pone.0350650.s008.tif]

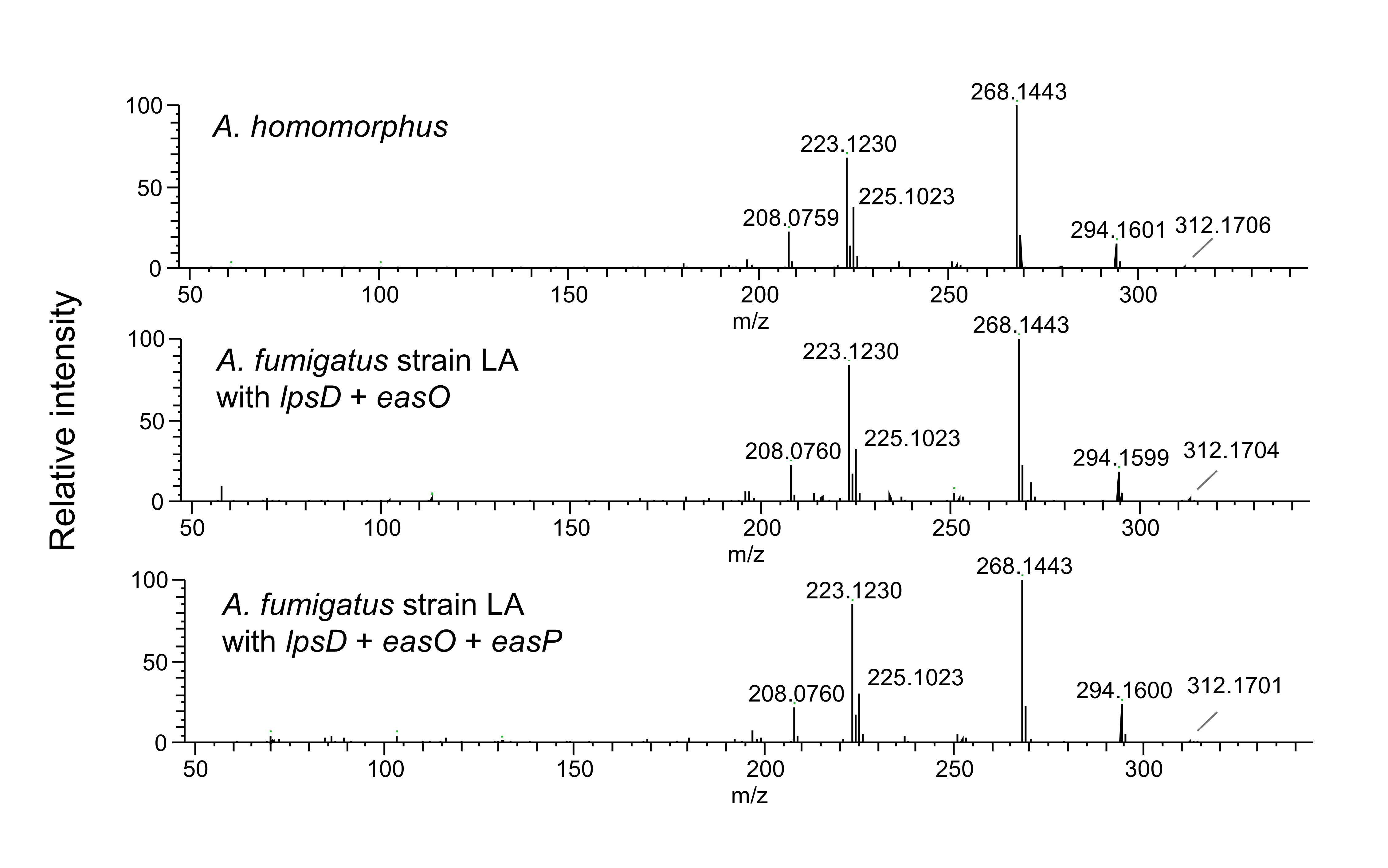

Supplement: S9 Fig — (TIF) [file pone.0350650.s009.tif]

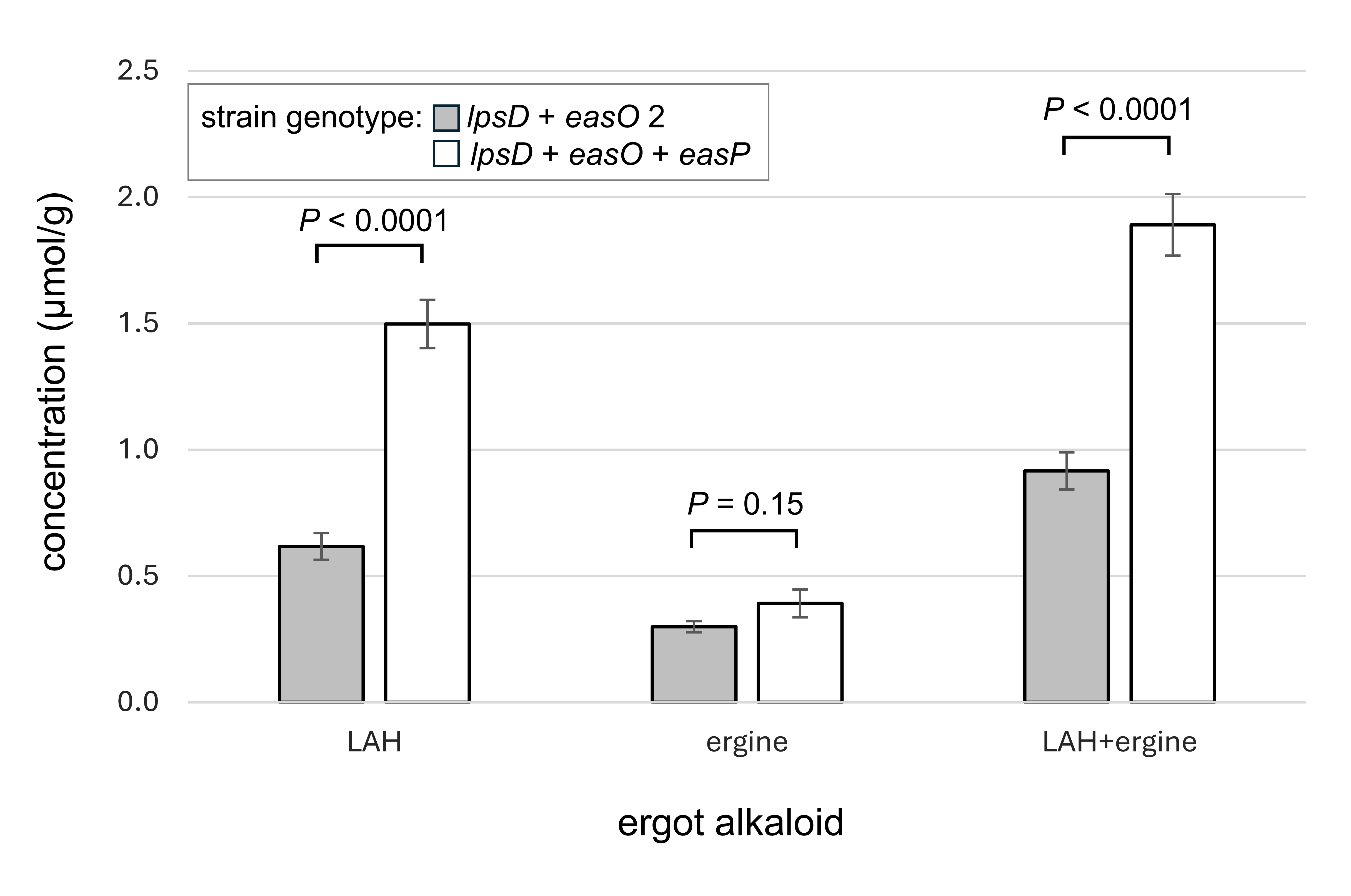

Supplement: S10 Fig — Error bars represent standard error. Quantities are derived from peak areas relative to those of an external standard curve or ergonovine and thus must be considered ‘relative to ergonovine’ as opposed to absolute. P values associated with one-way ANOVAs are shown for individual species of ergot alkaloids or a combination thereof. (TIF) [file pone.0350650.s010.tif]

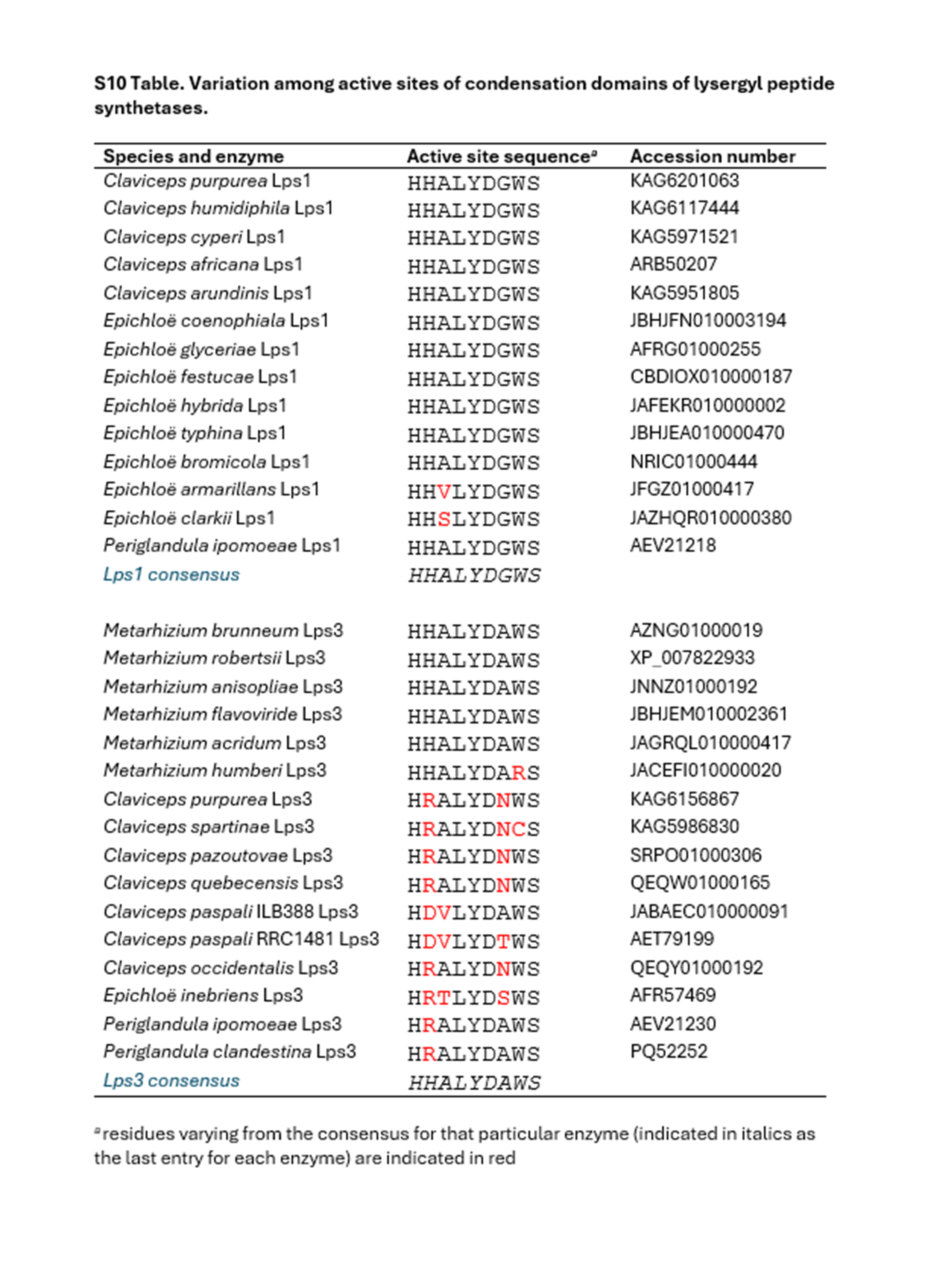

Supplement: S1 Table — (TIF) [file pone.0350650.s011.tif]
